# A comparative study of the efficacy of whether or not to preserve the joint capsule during PLIF surgery in patients with isthmic spondylolisthesis

**DOI:** 10.3389/fsurg.2025.1643744

**Published:** 2025-09-05

**Authors:** Jian Mi, Li Xu, Peng Yang, Tian-ci Fang, Li Ni, Hui-lin Yang, Quan Zhou, Feng Zhou

**Affiliations:** Department of Orthopaedics, The First Affiliated Hospital of Soochow University, Suzhou, China

**Keywords:** posterior lumbar interbody fusion (PLIF), isthmic spondylolisthesis (IS), joint capsule, spinal balance, adjacent segment degeneration (ASD)

## Abstract

**Purpose:**

The purpose of this study is to investigate the effect of joint capsule preservation during posterior lumbar interbody fusion (PLIF) on postoperative low back pain and long-term adjacent segment stability in isthmic spondylolisthesis (IS) patients.

**Methods:**

Group A: Forty-one patients received PLIF without preserving the joint capsule. Group B: Forty patients received PLIF with preserving the joint capsule. This study was randomly assigned, in which eligible patients were assigned to either group A (with joint capsule preservation) or group B (without joint capsule preservation). The radiographic outcomes were assessed via the lumbar lordosis (LL), segmental lordosis (SL), sacral slope (SS), pelvic incidence (PI), pelvic tilt (PT). The functional outcomes were evaluated via the Visual Analog Scale (VAS), Oswestry disability index (ODI), and reoperation rate. Metrics that describe the rate of degradation of adjacent segments include: the height of vertebral interval and the height of the upper intervertebral disc.

**Results:**

The sagittal balance parameters of the spine in the two groups were significantly improved after PLIF compared with those before surgery (*p* < 0.05). Compared with group A, the results of PI and PT in group B were significantly better than those in group A during the last follow-up (*p* < 0.05). Although the height of the upper intervertebral disc and vertebral interval did not show statistically significant results after surgery (*p* > 0.05), the height showed statistical differences during the last follow-up (*p* < 0.05).

**Conclusions:**

For patients with IS, we recommend that appropriate patients with IS should preserve the joint capsule as much as possible during PLIF, which is very helpful in preventing long-term degeneration of adjacent segments.

## Introduction

Isthmic spondylolisthesis (IS) is a common condition involving spinal surgery, and it is characterized by the dislocation of the isthmus, resulting in partial or complete slippage of the upper and lower vertebrae. In the current clinical treatment process, PLIF surgery has become the most important surgical method for treating IS. Surgical intervention can be successful in relieving symptoms, enabling earlier mobilization, and improving the quality of recovery and life (1–3). PLIF approach is a traditional lumbar approach, which can be entered through the posterior approach. It can directly remove the tissue compressing the nerve, such as intervertebral disc protrusion or osteophyte hyperplasia, and effectively relieve the symptoms of nerve compression (4). The implantation of fusion devices or bone grafts between the intervertebral spaces can promote intervertebral bone fusion, enhance spinal stability, and reduce postoperative pain and recurrence. This is a common treatment for patients with lumbar spondylolysis. PLIF allows for the full recovery of intervertebral height while maintaining posterior support structures and allowing for nerve decompression. Spinal fusion increases the risk of degeneration in adjacent segments by increasing mechanical stress and movement (5, 6).

Multiple studies have shown that after single segment PLIF surgery, the recovery of sagittal spinal balance is poor (7, 8), however, sagittal imbalance often leads to adjacent segment degeneration (ASD) (9). Although PLIF is the primary treatment for IS, it is also a risk factor for ASD (10–12). However, whether it is necessary to preserve the joint capsule while removing muscle tissue during PLIF surgery can improve the occurrence of postoperative ASD has become a question worthy of further discussion. Previous studies have reported complications of adjacent segment degeneration in patients after PLIF and analyzed some of the causes, but there have been no study specifically investigating the effect of capsule preservation on adjacent segment degeneration, so this study investigated and followed up for a long time to explore the benefits of preserving the joint capsule during PLIF for IS.

## Materials and methods

### Methods

The diagnostic criteria are as follows: 1. diagnosis of L4–L5 vertebral spondylolisthesis based on x-ray and CT imaging (x-ray and CT show anterior slippage of the L4 vertebral body and discontinuity of bone in the L4 vertebral arch isthmus). 2. The patient was diagnosed with L4–5 neurological symptoms after nerve block 3. PLIF surgery was performed on L4–L5, and the instruments and cage used during PLIF surgery were the same.

The exclusion criteria were as follows: 1. patients with lumbar spondylolisthesis caused by other reasons, including those with developmental disorders, acute fractures of posterior column structures such as pedicle and lamina, traumatic patients, and pathological patients with vertebral structure damage caused by bone diseases (tumors, infections, osteoporosis, etc.). 2. The patient has had lumbar spine disease in the past. 3. Patients who have undergone other spinal surgeries in the past. 4. Patients with severe underlying diseases. 5. Patients who are unable to complete a 3-year follow-up.

### Patient demographics

**Figure 1:**
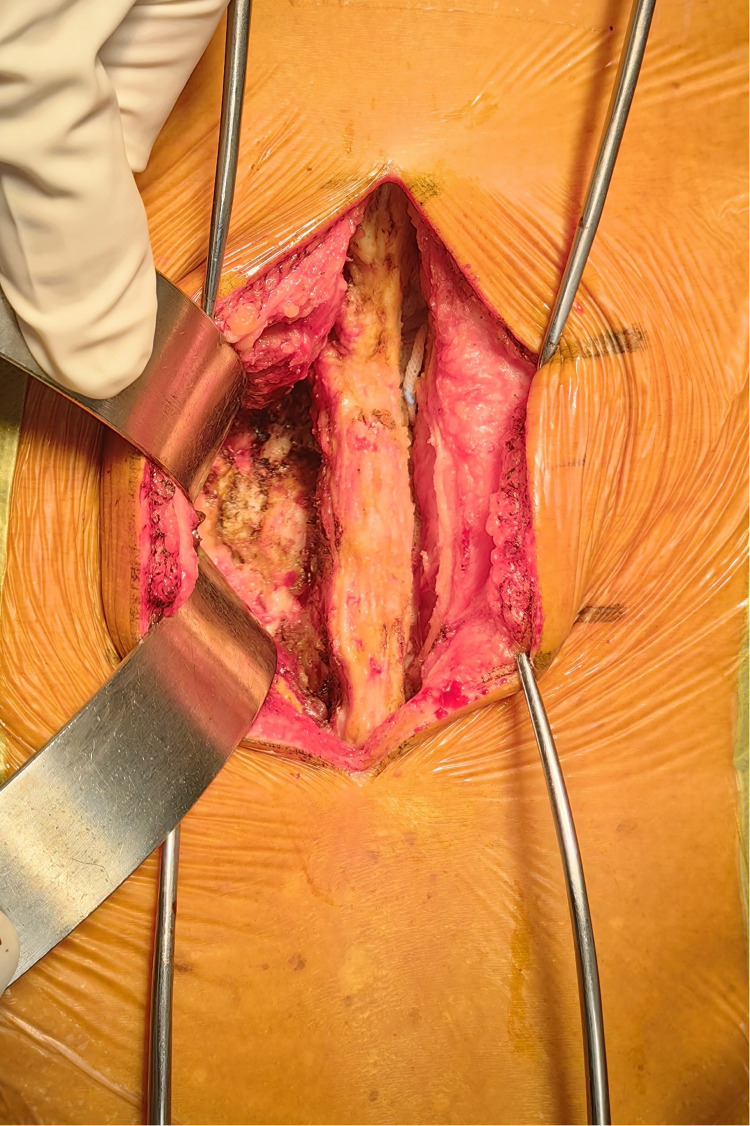
Pictures of the group that did not protect the joint capsule during PLIF surgery.

**Figure 2:**
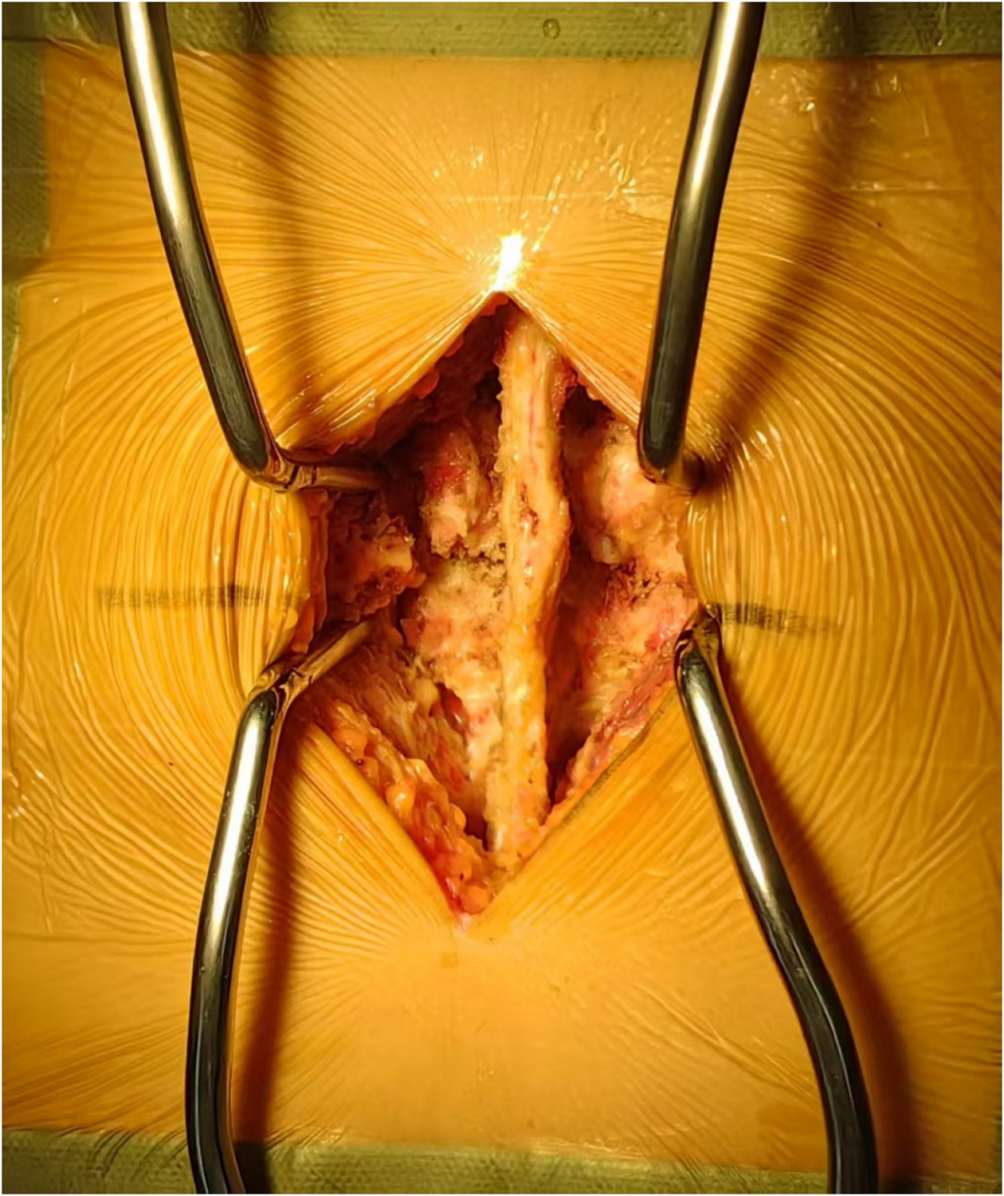
Pictures of the joint capsule group protected during PLIF surgery.

In line with the aforementioned inclusion and exclusion criteria, eighty-one patients with L4–L5 IS were recruited between July 2020 to June 2022 for our retrospective analysis. The patients were separated into Groups A and B. Group A: forty-one patients received PLIF at L4–L5 without preserving the joint capsule (Figure 1). Group B: forty patients received PLIF at L4–L5 with preserving the joint capsule (Figure 2). During our follow-up process, all participants signed informed consent forms. All patients selected for this experimental group have undergone surgery by the same group of doctors.

To control confounding bias, the patients included in this experiment underwent early postoperative rehabilitation (1–2 weeks) with bed rest management, progressive lower limb movement, and pain control. During the mid-term (2–6 weeks), gradually practice getting out of bed and walking while wearing waist protection; After postoperative outpatient follow-up, it is necessary to evaluate the fusion situation based on imaging and adjust the rehabilitation plan accordingly, including low-intensity aerobic exercise for lumbar and back muscle training. According to the classification of labor intensity levels I-IV, the population included in the experiment only engaged in light to moderate labor after surgery.

### Surgical technique

**Figure 3:**
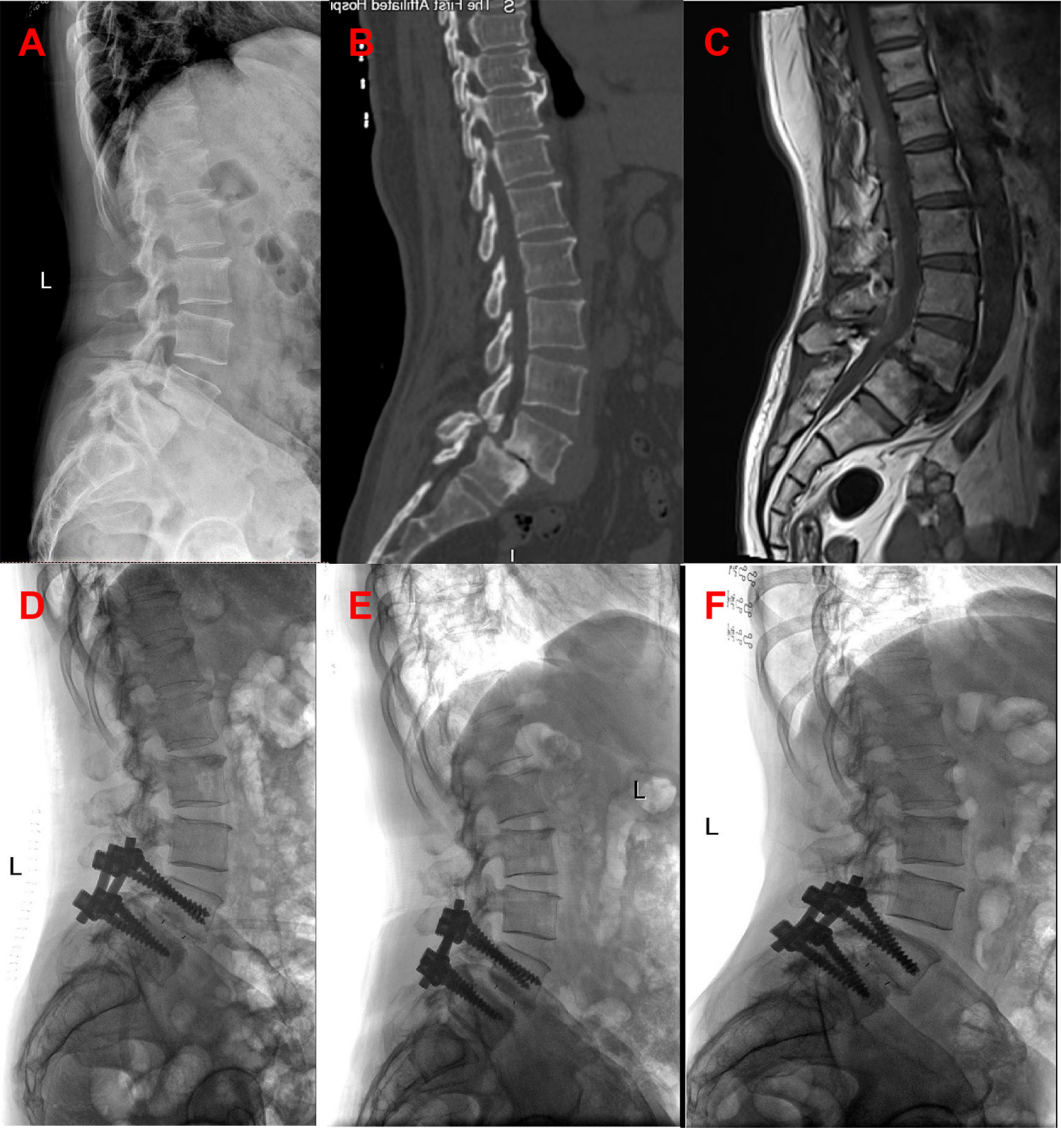
Patients in group A were preoperative x-ray **(A)**, preoperative CT **(B)**, preoperative MRI **(C)**, postoperative x-ray, postoperative x-ray 3 months later, and the last follow-up.

**Figure 4:**
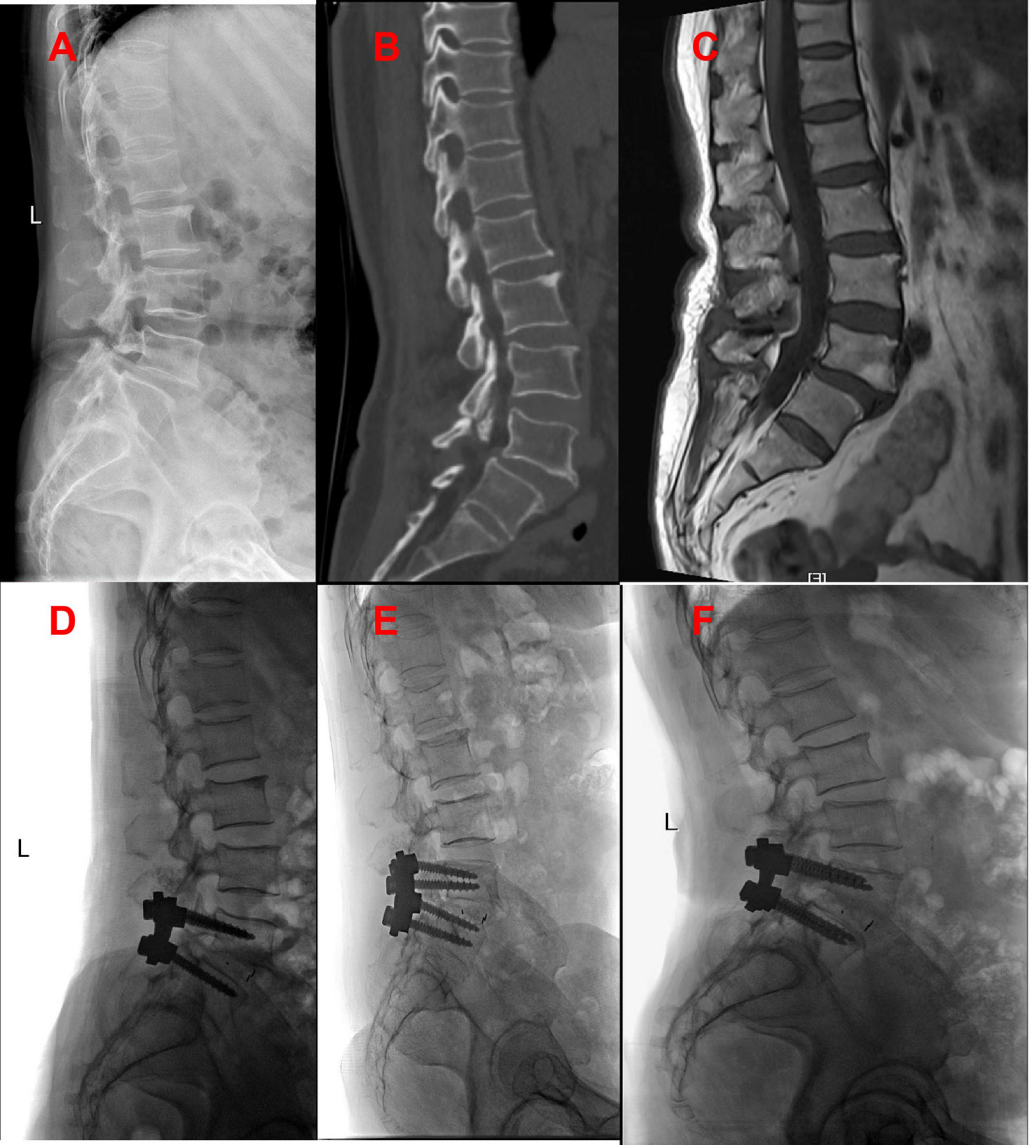
Patients in group B were preoperative x-ray **(A)**, preoperative CT **(B)**, preoperative MRI **(C)**, postoperative x-ray, postoperative x-ray 3 months later, and the last follow-up.

All surgeries were performed by two experienced orthopedic surgeons. Use C-arm perspective to determine the entry point of the affected segment. Posterior midline incision, dissection of muscle tissue, exposure of vertebral plate and articular process. Under the guidance of C-arm x-ray fluoroscopy, two pedicle screws were routinely implanted into the L4 and L5 vertebrae (group A patients with exposed joint capsule, the preservation of the joint capsule is mainly achieved through fine microsurgical techniques, including 1. carefully separating and preserving the soft tissue around the joint capsule before the implantation of the intervertebral fusion device; 2. use small joint partial resection technique (only remove the inner 1/3 of the articular process) to avoid damaging the attachment point of the joint capsule; 3. during the operation, the integrity of the joint capsule was confirmed through direct visualization and intraoperative C-arm examination. Then, part of the lamina is removed to expand the spinal canal and expose the dura mater and nerve roots. The bone spurs, ligaments (such as the yellow ligament), or protruding intervertebral disc tissue that compress the nerves are cleared. Next, the dural sac and nerve roots are gently stretched to expose the intervertebral space. The annulus fibrosus is incised, and the nucleus pulposus and cartilage endplates are thoroughly removed. The cartilage endplate is scraped until it bleeds, avoiding damage to the bony endplate that could cause collapse. Finally, an intervertebral fusion cage filled with bone grafts is implanted into the intervertebral space to restore the intervertebral height and physiological curvature (Figures 3, 4).

### Clinical evaluation

Perioperative parameters, including hospitalization days, blood loss, duration of surgery, bone mineral density (BMD) and body mass index (BMI) were recorded. All patients were surveyed before surgery, as well as 3 days, 3 months, and 3 years after surgery: Through VAS scores, the subjective pain experience of patients in this experiment at different time points was quantified (13). Meanwhile, this experiment also assessed the functional limitations of patients at different time points through the ODI scores (14).

### Radiographic evaluation

**Figure 5:**
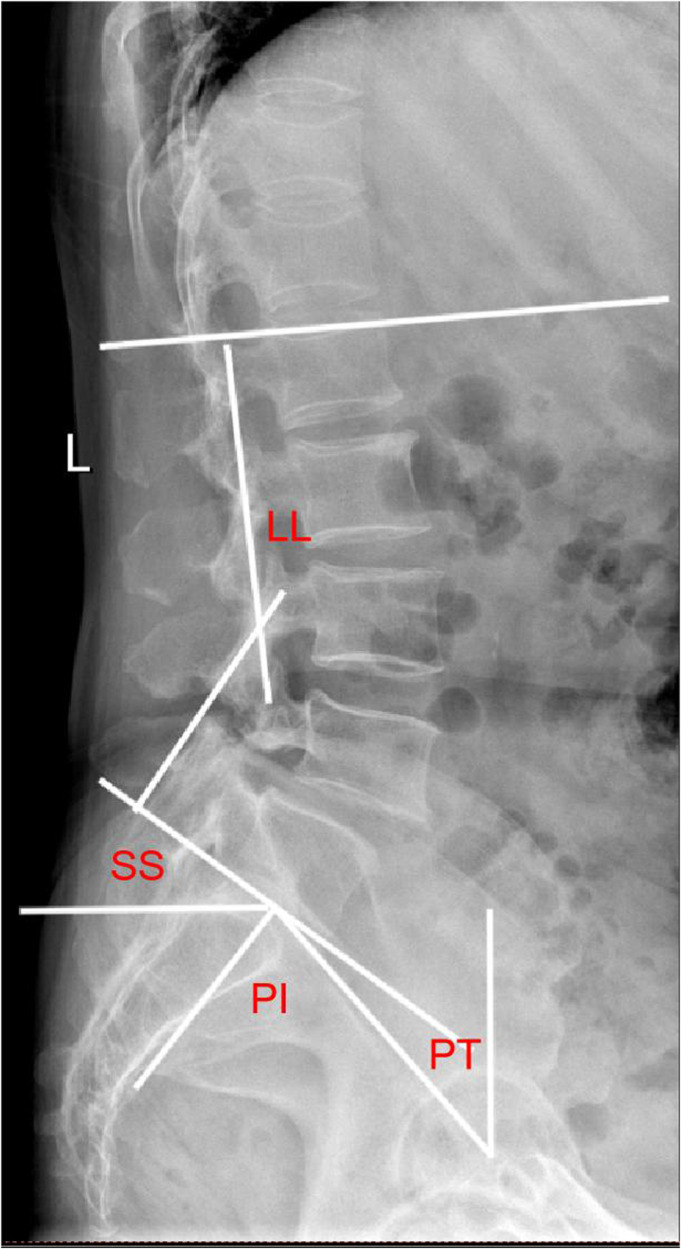
Plain lateral radiograph for measuring radiographic parameters; SL: segmental lordosis; LL: lumbar lordosis; SS: sacral slope; PI: pelvic incidence; PT: pelvic tilt.

The EOS system assesses changes in lumbar lordosis (LL), segmental lordosis (SL), pelvic incidence (PI), sacral slope (SS), pelvic tilt (PT) (Figure 5). LL is defined as the angle between the upper end plates of L1 and S1. PI is the line connecting the midpoint of the sacral upper articular plate to the center of the femoral head. PT is the angle between the line connecting the midpoint of the superior sacral plate to the center of the femoral head and the vertical line. SS is the angle between the superior articular plate of the sacrum and the horizontal line. SL is the angle between the upper end plate of L5 and the lower end plate of the S1 vertebrae. All patients underwent pre-posterior imaging 3 days after surgery, 3 months after surgery and at 3 years after surgery.

### Statistical methods

The measurement data is expressed as mean ± standard deviation. In this experiment, paired sample *t*-test and chi square test were used to process the data. *P* < 0.05 is considered statistically significant.

## Results

### Demographics

The basic information of the patients in both groups is in Table 1. Group A included 11 males and 30 females, with an average age of 59.86 ± 6.143 years. Group B included 7 males and 33 females, with an average age of 60.05 ± 6.80 years. The average length of stay in group A was 13.17 ± 2.31 days, and that in group B was 13.50 ± 3.81 days. The average BMD in group A was −1.43 ± 0.71, and the average BMD in group B was −1.51 ± 0.80. The average BMI of group A was 22.41 ± 2.04 and that of group B was 22.67 ± 2.02. There were no significant differences in terms of age, gender, BMD, BMI, surgical blood loss, Meyerding slip grades, and follow-up time between the two groups (*p* > 0.05).

**Table 1:** Demographic data of all patients.

Baseline	Full sample	Group A	Group B	*P* value
Number of patients	81	41	40	
Age (years)	59.97 ± 6.44	59.86 ± 6.143	60.05 ± 6.804	0.213
Gender (male/female)	18/63	11/30	7/33	0.828
BMD (T-score)	−1.47 ± 0.75	−1.43 ± 0.71	−1.51 ± 0.80	0.873
BMI (kg/m^2^)	22.54 ± 2.03	22.41 ± 2.04	22.67 ± 2.02	0.722
Comorbidities (n)				
Hypertension	34/47 (41.98%)	16/25 (39.02%)	18/22 (45.00%)	0.586
Diabetes	18/63 (22.22%)	8/33 (24.24%)	10/30 (25.00%)	0.553
Hyperlipidemia	25/56 (30.86%)	14/27 (34.14%)	11/29 (27.50%)	0.517
Smoking	18/63 (32.04%)	10/31 (28.27%)	8/32 (18.03%)	0.653
Alcoholism	10/71 (12.35%)	5/36 (12.20%)	5/35 (14.29%)	0.967
Duration of surgery (min)	111.43 ± 8.21	111.65 ± 8.52	111.20 ± 7.98	0.849
Surgical blood loss (ml)	156.91 ± 19.58	162.07 ± 20.70	151.62 ± 17.03	0.316
LOS (days)	13.33 ± 3.13	13.17 ± 2.31	13.50 ± 3.81	0.090
Follow-up (months)	40.76 ± 3.97	40.48 ± 4.23	41.05 ± 3.70	0.166

BMD, bone mineral density; BMI, body mass index; LOS, length of stay.

### Clinical outcomes

The ODI and VAS scores of both groups were shown in Table 2. Compared with the preoperative results, the VAS and ODI scores 3 days after operation and 3 months after operation showed no differences in all two groups (*p* >0.05). However, Group B showed better VAS and ODI scores than Group A at 3 years after operation (*p* <0.05).

### Radiological results

The Radiological results of both groups were shown in Tables 3 and 4.

**Table 2:** Functional impairment score and pain index score.

Parameter	Record time	Group A	Group B	*P* value
VAS	Preop	7.22 ± 0.82	7.33 ± 0.89	0.400
	Postop 3d	2.95 ± 0.71	2.76 ± 0.54	0.401
	Postop 3m	2.95 ± 0.67	2.93 ± 0.47	0.056
	Postop 3y	3.19 ± 1.12	2.80 ± 0.69	**0**.**003**
ODI	Preop	66.56 ± 4.20	68.50 ± 4.97	0.123
	Postop 3d	26.07 ± 2.68	26.30 ± 2.59	0.751
	Postop 3m	26.53 ± 2.57	26.27 ± 2.22	0122
	Postop 3y	29.14 ± 4.19	25.22 ± 3.11	**0**.**018**

Preop, preoperation; Postop 3d, three days after operation; Postop 3m, three months after operation; Postop 3y, three years after operation; ODI, Oswestry disability index; VAS, visual analog scale. Font bold indicates statistical difference, *P* < 0.05.

**Table 3:** Sagittal balance parameters.

Parameter	Record time	Group A	Group B	*P* value
SL	Preop	17.51 ± 3.60	17.45 ± 4.21	0.298
	Postop 3d	24.22 ± 3.78	24.52 ± 2.59	0.076
	Postop 3m	23.17 ± 3.78	24.05 ± 2.95	0.368
	Postop 3y	24.24 ± 4.51	25.52 ± 3.67	0.374
LL	Preop	37.97 ± 3.14	38.27 ± 4.14	0.057
	Postop 3d	44.90 ± 4.24	45.20 ± 4.73	0.156
	Postop 3m	43.56 ± 4.67	45.05 ± 4.63	0.405
	Postop 3y	41.41 ± 4.27	44.25 ± 4.17	0.908
PI	Preop	54.09 ± 4.05	54.95 ± 4.28	0.680
	Postop 3d	58.65 ± 4.00	55.08 ± 5.22	0.293
	Postop 3m	56.29 ± 4.01	54.83 ± 5.66	0.092
	Postop 3y	51.24 ± 3.64	52.60 ± 6.07	**<0**.**001**
SS	Preop	37.46 ± 4.16	38.17 ± 5.38	0.072
	Postop 3d	37.37 ± 4.24	36.37 ± 5.07	0.053
	Postop 3m	37.31 ± 4.09	35.92 ± 4.84	0.060
	Postop 3y	34.51 ± 5.61	34.72 ± 6.53	0.291
PT	Preop	18.66 ± 4.70	19.55 ± 3.77	0.072
	Postop 3d	15.92 ± 5.03	16.02 ± 4.79	0.593
	Postop 3m	16.02 ± 5.07	16.02 ± 5.07	0.482
	Postop 3y	18.88 ± 5.63	17.35 ± 3.89	**0**.**018**

Preop, preoperation; Postop 3d, three days after operation; Postop 3m, three months after operation; Postop 3y, three years after operation; LL, lumbar lordosis; SL, segmental lordosis; PI, pelvic incidence; PT, pelvic tilt; SS, sacral slope. Font bold indicates statistical difference, *P* < 0.05.

At the final follow-up, the sagittal balance data was significantly better than preoperative, indicating that PLIF surgery has a good therapeutic effect on patients with lumbar spondylolysis. In both groups, group B had slightly better SS, LL, PI, SL and PT 3 months after operation than group A (*p* >0.05). This finding was particularly evident at 3 years after operation, the PI and PT in Group B were significantly better than those in Group A (*p* < 0.05).

According to Table 4 of the results, the height of vertebral interval and the height of the upper intervertebral disc were not statistically significant before operation, three days after operation, 3 months after operation (*p* >0.05). Over time, the lumbar intervertebral disc will slowly degenerate, and the indicators related to intervertebral disc degeneration in group A and Group B, including the height of vertebral interval and the height of the upper intervertebral disc, were significantly different. The average value of the height of vertebral interval in group A was 12.55 ± 1.99, and that in group B was 13.82 ± 2.58 3 years after the operation (*p* < 0.05). There was no significant difference in the grading of lumbar spondylolisthesis between the two groups before operation, three days after operation, 3 months after operation, but the data from the three-year follow-up after operation showed significant significance (*p* < 0.05).

**Table 4:** Related parameters of lumbar degeneration.

Parameter	Record time	Group A	Group B	*P* value
The height of vertebral interval	Preop	12.97 ± 2.31	13.37 ± 2.26	0.994
	Postop 3d	13.69 ± 2.16	14.45 ± 2.41	0.190
	Postop 3m	13.67 ± 2.15	14.29 ± 2.30	0.357
	Postop 3y	12.55 ± 1.99	13.82 ± 2.58	**0**.**045**
The height of the upper intervertebral disc	Preop	15.34 ± 3.86	19.71 ± 5.40	0.053
	Postop 3d	15.60 ± 5.58	18.87 ± 6.78	0.257
	Postop 3m	15.70 ± 5.06	18.70 ± 6.25	0.222
	Postop 3y	20.12 ± 5.43	20.95 ± 7.25	**0**.**039**
Meyerding grade	Preop	2.63 ± 0.70	2.70 ± 0.69	0.740
	Postop 3d	1.07 ± 0.26	1.05 ± 0.22	0.951
	Postop 3m	1.12 ± 0.33	1.07 ± 0.27	0.160
	Postop 3y	1.41 ± 0.55	1.25 ± 0.44	**0**.**005**

Preop, preoperation; Postop 3d, three days after operation; Postop 3m, three months after operation; Postop 3y, three years after operation. Font bold indicates statistical difference, *P* < 0.05.

## Discussion

In the current study, we reviewed 81 patients who underwent instrumental lumbar fusion at L4–L5 with an exposed and non-exposed capsule during nail exposure, with a mean follow-up of 40.7 months. The results showed that patients who preserved the joint capsule were better than those who did not preserve the joint capsule in terms of recovery of some sagittal balance parameters after surgery, and the incidence of postoperative lumbar disc degeneration was lower.

IS is caused by a fracture or defect in the isthmus of the lumbar vertebral arch, involving a vertebra moving forward relative to an adjacent vertebra. In clinical, conservative treatment with anti-inflammatory and analgesic drugs is first considered for IS. If the symptoms do not improve and worsen over time, affecting their daily lives, PLIF surgery will become the preferred treatment option (15, 16). Many studies have shown that PLIF can significantly alleviate patients’ symptoms and restore their quality of life compared to non-surgical treatment (17–19). PLIF surgery can effectively relieve patients’ pain symptoms and improve their quality of life by eliminating nerve root compression and restoring spinal stability. In addition, the surgery also helps restore spinal function and maintain the normal anatomical structure of the spine. However, PLIF surgery itself may cause changes in local anatomical structures, such as increased stress in the fused segment and degenerative changes in adjacent joints (20). How to solve the degeneration of adjacent joints has been a long-standing problem in clinical practice. In PLIF surgery, few people pay attention to whether the joint capsule needs to be removed when the incision is exposed. In this study, we investigated whether the joint capsule was preserved during PLIF surgery for the treatment of patients with lumbar spondylolisthesis with isthmic fissure, with a particular focus on the restoration of sagittal balance and its effect on lumbar disc degeneration.

The joint capsule is composed of dense connective tissue, rich in collagen fibers, and has tensile strength. The fiber layer of some joints fuses with ligaments to enhance stability (21–23). To our knowledge, this study is the first to use meta-analyses and systematic reviews to demonstrate a higher level of evidence for the improvement of joint capsule-related complications after PLIF. Intraoperative preservation of the joint capsule helps to maintain the original structure and function of the joint, thereby avoiding excessive tissue damage after surgery and reducing the area of soft tissue damage. This study has shown that preservation of the joint capsule reduces VAS and ODI. Because the joint capsule is an important stabilizing structure, it protects the biomechanical properties of the joint and avoids excessive tissue traction and friction after surgery, thereby reducing the duration of postoperative pain. Postoperative ODI scores were lower, especially in scores assessing motor function, walking ability, and daily activities. The preservation of the joint capsule allows for a better recovery of the patient's mobility in the early postoperative period, thereby reducing the disability score due to motor dysfunction. The postoperative VAS score will also be reduced accordingly, which means that the patient's subjective pain is reduced and the corresponding quality of life of the patient will be improved. This result may be related to the biomechanical function of the joint capsule, which is rich in mechanoreceptors and can maintain the dynamic stability of the lumbar spine and reduce the stimulation of abnormal stress on the nerve root. In addition, the preservation of the joint capsule may reduce abnormal movement of the articular process after surgery, thereby reducing the occurrence of chronic pain.

The pelvic entry angle is an anatomical parameter that is not affected by changes in posture. It increases gradually between the ages of 4 and 18, with little fixed change in adulthood (24), and plays an important role in sagittal balance as well as in the process of lumbar degeneration. Compensatory mechanisms for sagittal imbalance can occur in the spine, pelvis, and lower extremities, and they often exhibit varying degrees of compensation depending on the degree of imbalance, spinal mobility, or muscle strength. For lumbar lordosis loss due to lumbar degeneration, hyperextension of adjacent segments of the degenerative segment is a very common compensatory mechanism. There are studies showing that patients with lower back pain have vertebral body edge osteophyte formation and ligament calcification (25). More proximal lumbar compensatory hyperextension can effectively shift the center of gravity of the upper spine backward and maintain overall sagittal balance. However, the stress of adjacent segments also increases resulting in an increased risk of adjacent segment degeneration (ASD). In this study indicated that post PLIF, IS patients achieved better reduction of the slipped vertebra and better recovery of the sagittal balance of the entire spine. Pelvic parameters include PI, PT, LL, SL and SS. PT is a characteristic of pelvic rotation, and the standard value is about 13° ± 6° (26). Sung-Soo et al. reported that there is a strong correlation between PT and VAS and DOI scores (27). In our study, there was a statistical difference in PT between the two groups in more than three years of the last follow-up. In Group A, PT significantly increased three years after surgery, while VAS and ODI also correspondingly increased. In Group B, the opposite situation occurred, which may explain why there were differences in ODI and VAS scores between the two groups. In some previous studies, Nakashima et al. found high PI, not LL (28), a significant risk factor for early-onset ASD. In a retrospective analysis of Alentado et al. (29), SL and LL were not significant risk factors for ASD. In this study, this viewpoint has been fully confirmed. Previous studies have supported that smaller PI can lead to loss of upper lumbar curvature and development of scoliosis, ultimately resulting in adjacent intervertebral disc degeneration over time and under adverse living conditions (30–32). We observed no obvious differences in the sagittal balance parameters at discharged from hospital and 3 months postoperatively between the two groups. However, in more than one year of the last follow-up, The PI of group B is significantly better than that of group A. The PI in group A will be significantly smaller than that in group B after prolongation of time. The results of this study indicate that in Group A, there is a loss of lumbar curvature, which subsequently leads to lumbar instability, and ultimately, a higher probability of developing lumbar degeneration. The retention of the joint capsule helps to maintain the stability of the facet joints and reduce the abnormal lumbar spine sequence caused by surgical damage. However, in some patients with resection, PI-PT mismatch may be related to the increase of small joint mobility after joint capsule resection, leading to a decrease in compensatory lumbar lordosis.

This study found that patients who preserved the joint capsule were more likely to recover sagittal balance after surgery, its integrity can effectively support the stability of intervertebral discs, articular surfaces and ligaments, and reduce the damage to the posterior structures of the spine during surgery. PLIF surgery has been proven effective in treating lumbar spondylolisthesis, but it is important for clinical doctors to address postoperative complications such as ASD. The most common manifestations of ASD include disc degeneration, segmental instability, facet joint hyperplasia, and spinal stenosis. ASD-free survival rates at 5 and 10 years postoperatively were 93.8% and 90.1%, respectively (33). Therefore, reducing the occurrence of ASD is not only beneficial to postoperative recovery, but also beneficial to improve the quality of life of patients in the long term. Menezess-Reis et al. reported that lumbar lordosis is flat and has a higher frequency of disc degeneration at L4/5 than a long, curved lumbar spine (34). This was also directly confirmed by our study, which showed that lumbar lordosis was better at 3 months postoperatively in the non-capsule group than in the non-capsule exposure group. This result was more pronounced after three years of follow-up. However, there is more controversy about the impact of disc space height recovery on the development of ASD. It is thought that increased intervertebral space height increases the incidence of ASD. Liu et al. (34) proposed that an increase in intervertebral space height significantly alters the mechanical properties of facet joints and may therefore lead to facet joint subluxation. In contrast, Tang et al. (35) pointed out that L3/4 has the highest intervertebral disc pressure, L4/5 has significantly reduced intervertebral disc height, and intervertebral disc pressure is the lowest. He was credited with the experiment that intradiscal pressure gradually increases with decreasing disc space height. Our data supports the conclusions drawn by Tang et al. With the extension of follow-up time, the disc space height of the unexposed capsule group was significantly higher than that of the exposed capsule group after the PLIF operation, and reasonable restoration of cone height was helpful to restore the normal biomechanical state of the spine, reduce the pressure of adjacent segments, and thus reduce the risk of ASD. Related studies have shown that the decrease of lumbar intervertebral space height leading to ASD is associated with the following related factors. First of all, a decrease in intervertebral disc height weakens its elastic cushioning function, leading to ligament laxity or compensatory hypertrophy, and a decline in spinal dynamic stability. This can trigger micro-movements or abnormal activities, accelerating degeneration. Second, a reduced disc height also diminishes the nucleus pulposus's diffusion capacity, causing metabolic waste accumulation and inadequate nutrient supply, which further exacerbates dehydration, fibrosis, and annulus fibrosus rupture of the nucleus pulposus. Last, a decrease in lumbar intervertebral space directly reduces the volume of the intervertebral foramen, potentially compressing nerve roots and causing pain and inflammation. Chronic irritation can lead to perineural fibrosis and adhesion (36). Our study showed that the height of the adjacent upper intervertebral disc in the exposed capsule group decreased significantly compared with the non-exposed capsule group with the increase of postoperative years, which increased the pressure of the intervertebral disc and affected the nutrient supply of the intervertebral disc (37), thereby causing lumbar disc degeneration in adjacent segments. Therefore, it can be seen that the restoration of normal intervertebral space height and upper disc height is extremely important measures to prevent and reduce the occurrence of adjacent segment degeneration after lumbar fusion. The Meyerding classification of lumbar spondylolisthesis is significantly correlated with the degree of degeneration: patients with grade I–II spondylolisthesis typically exhibit early degenerative features, such as mild reduction in intervertebral disc height and mild hypertrophy of facet joints; Patients with grade III or above spondylolisthesis often experience more severe multi segment degeneration, including intervertebral disc dehydration, endplate Modic changes, and facet joint osteoarthritis. During the final follow-up after surgery, there was a significant difference between the two groups, indicating that the lumbar degeneration in the joint capsule protection group was significantly better than that in the non-joint capsule protection group.

There is one more point that needs to be further supplemented regarding joint capsule protection: First, Young patients (<50 years old) with mild degeneration of facet joints (evaluated according to MRI or CT grading) have better joint stability; Second, For patients with single segment slippage (Meyerding I–II degree), preserving the joint capsule may better maintain segment stability; Last, For patients with obvious mechanical pain in the lower back before surgery but without severe neurological symptoms, preserving the joint capsule may reduce the risk of adjacent segment degeneration after surgery. However, for patients with severe degenerative changes (Pfirrmann grade ≥3) or significant hypertrophy of facet joints, preserving the joint capsule may increase the risk of persistent postoperative pain, and caution should be exercised to weigh the pros and cons. This provides more reference opinions for clinical surgery.

However, there are some limitations to this study, firstly, it was not chosen at random in the study design. Non-random assignment may affect the quantitative evaluation-non-functional questionnaire based on the patient's subjective evaluation. The second is that the number of follow-up people is small and the follow-up time is limited, which may have an impact on the long-term stability of the study results. Future researches will improve these deficiencies to further validate the long-term effects of preserving the joint capsule in patients with lumbar spondylolisthesis with isthmic fissures and its specific effect on lumbar disc degeneration. Future researches can still be explored in the following directions: firstly, it is recommended to conduct long-term follow-up studies (≥5 years) with multiple centers and large sample sizes, focusing on the impact of joint capsule preservation on adjacent segment degeneration, long-term functional prognosis, and quality of life; Furthermore, exploring the application value of new surgical techniques such as navigation assisted or robotic surgery in accurately preserving joint capsules; Finally, it is recommended to conduct a cost-benefit analysis to evaluate the clinical application value of this technology from the perspective of health economics. These studies will help develop more precise individualized treatment plans and provide more comprehensive evidence-based support for clinical practice. In conclusion, this study showed that the preservation of the joint capsule during PLIF surgery has a positive effect on the recovery of sagittal balance and lumbar disc degeneration in patients with lumbar spondylolisthesis with isthmus fissure, which provides a valuable basis for the optimization of clinical surgical strategies.

## Conclusion

For patients with IS, PLIF surgery can achieve prominent sagittal balance and functional outcomes postoperatively. In long-term follow-up, the preservation of the joint capsule has a clear advantage in the restoration of sagittal balance and the reduction of adjacent segment degeneration after surgery, Therefore, we recommend that the superior joint capsule should be preserved as much as possible for IS patients undergoing PLIF surgery.

## Data Availability

The raw data supporting the conclusions of this article will be made available by the authors, without undue reservation.
